# Germline *BRCA*, chemotherapy response scores, and survival in the neoadjuvant treatment of ovarian cancer

**DOI:** 10.1186/s12885-020-6688-8

**Published:** 2020-03-04

**Authors:** Yong Jae Lee, Hyun-Soo Kim, John Hoon Rim, Jung-Yun Lee, Eun Ji Nam, Sang Wun Kim, Sunghoon Kim, Young Tae Kim

**Affiliations:** 1 Department of Obstetrics and Gynecology, Institute of Women’s Life Medical Science, Yonsei University College of Medicine, 50–1 Yonsei-ro, Seodaemun-gu, 03722 Seoul, Republic of Korea; 2 Department of Pathology, Severance Hospital, Yonsei University College of Medicine, Seoul, South Korea; 3 Department of Laboratory Medicine, Severance Hospital, Yonsei University College of Medicine, Seoul, South Korea

**Keywords:** Ovarian cancer, Neoadjuvant chemotherapy, Germline *BRCA*, Chemotherapy response scores

## Abstract

**Background:**

To analyze the effects of *BRCA1/2* mutations on chemotherapy response scores (CRS) and survival in a cohort of patients with advanced-stage ovarian cancer who were treated with neoadjuvant chemotherapy (NAC) followed by interval debulking surgery (IDS).

**Methods:**

We retrospectively reviewed the medical records of 169 high-grade serous ovarian cancer patients who underwent a germline *BRCA1/2* test and received three cycles of NAC at the Yonsei Cancer Center from 2006 to 2018. Chemotherapy response scores were compared in patients with and without *BRCA1/2* mutations. The effects of *BRCA1/2* mutations and CRS on survival were evaluated.

**Results:**

*BRCA1/2* mutations were detected in 47 (28.1%) of the 169 patients. Overall, 16 (34.0%) patients with *BRCA1/2* mutations had a CRS 3 to chemotherapy compared to scores of 43 in patients (35.2%) without a mutation. Response scores of 3 in patients with *BRCA1/2* mutations were not significantly associated with either improved progression-free survival (PFS) (*P* = 0.949) or overall survival (OS) (*P* = 0.168). However, CRS 3 in patients without *BRCA* mutations was significantly associated with both improved PFS (*P* = 0.030) and OS (*P* = 0.039). In patients with CRS1/2, carriers of *BRCA1/2* mutations had better PFS (*P* = 0.0344) and OS (*P* = 0.043) than wild-type *BRCA* genotype patients.

**Conclusion:**

In ovarian cancer patients treated with NAC, CRS did not predict survival for *BRCA 1/2* mutation carriers but did for *BRCA* wild-type patients.

## Background

Primary cytoreductive surgery followed by platinum-based doublet chemotherapy has been considered standard treatment for advanced-stage ovarian cancer. However, several randomized clinical trials showed that their survival outcomes and postoperative morbidity and mortality after neoadjuvant chemotherapy (NAC) followed by interval debulking surgery (IDS) were at least as good as the results for patients who primary cytoreductive surgery [1–4]. Recently, NAC followed by IDS has become an alternative treatment for advanced-stage ovarian cancer patients.

Bohm et al. proposed a three-tiered histopathologic scoring system for grading the response to NAC [5]. A three-tiered chemotherapy response scores (CRS) system was applied to the omental tissue sections and correlated with progression-free survival (PFS) [5, 6]. As a consequence, an increasing body of research has addressed whether CRS 3 can be used as a surrogate marker, similar to the way the pathologic complete response (pCR) is used in breast cancer, in the prognosis for patients with advanced-stage ovarian cancer treated with NAC followed by IDS. However, platinum-based doublet chemotherapy remains the standard of care for advanced-stage ovarian cancer patients who are treated with NAC followed by IDS, regardless of their response to chemotherapy.

Mutations in *BRCA1* and *BRCA2* have been recognized as a predictor of advanced-stage ovarian cancer susceptibility and a prognostic factor [7–9]. Compared with wild-type *BRCA* genotype patients, patients with advanced-stage ovarian cancer and *BRCA1/2* mutations have been reported to have higher clinical response rates to platinum-based chemotherapy [10–12]. Therefore, there are unanswered questions about whether the higher CRS by carriers of the *BRCA1/2* germline mutations represents a better prognosis. In triple-negative breast cancers treated with NAC, several studies have tried to identify the relationships between germline *BRCA1/2* mutations, response rates, and prognoses [13, 14]. These studies have shown that patients with *BRCA1/2* mutations had superior response rates, and response rate was a weaker predictor of disease-free survival rates compared with wild-type *BRCA1/2* genotype patients.

In this study, we analyzed the extent to which CRS depended on germline *BRCA1/2* mutations, whether CRS correlates with platinum-based chemotherapy, and whether CRS has an impact on survival outcomes in patients with and without germline *BRCA1/2* mutations.

## Methods

### Study populations

We retrospectively reviewed the medical records of 326 patients with pathologically confirmed ovarian cancer who from 2006 to 2018 received NAC at the Yonsei Cancer Center, Seoul, South Korea. Patients with stage III or IV ovarian carcinoma who received three cycles of NAC followed by IDS were included in the study.

**Fig. 1:**
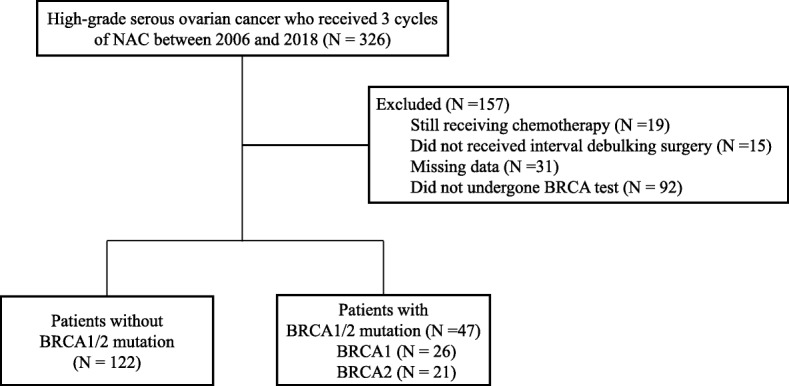
Flow diagram of the study population. NAC, neoadjuvant chemotherapy

The exclusion criteria were as follows: Patients still receiving chemotherapy at the time of data analysis (*N* = 19); patients who had not received IDS after NAC (*N* = 15); patients for whom data was missing (*N* = 31); and, lastly, patients who had not undergone a germline *BRCA* test (*N* = 92). After this review, 169 patients met our criteria. Of these, 122 patients had the wild-type *BRCA* genotype, and 47 had *BRCA1* or *BRCA2* mutations (Fig. 1).

### Treatment

Most patients received taxane (paclitaxel, docetaxel) and platinum (carboplatin) combination chemotherapy and some patients received paclitaxel, carboplatin and bevacizumab combination chemotherapy. Other treatments such as radiation or endocrine therapy were not performed before surgery. Determination of which patients required NAC was based on initial imaging studies that showed high tumor dissemination with high risk of postoperative comorbidities and poor performance status, or optimal cytoreduction surgery (residual disease measuring 1 cm or less) was unsuitable because of a high tumor burden [predictive index value (PIV) ≥ 8] [15]. For diagnostic laparoscopy, the degree of tumor burden was determined with the PIV [16]. For IDS, all patients underwent surgery with the intent to achieve complete resection with no residual tumor. Standard surgical procedures included hysterectomy, bilateral oophorectomy, omentectomy, pelvic/para-aortic lymph node dissection, and appendectomy. Radical surgery included more aggressive procedures such as liver resection or bowel resection than those who underwent standard surgical procedures [17]. Subsequently, additional cycles of adjuvant chemotherapy were administered to complete a total of six cycles at the discretion of the treating physician. Surgical complexity was classified as low, intermediate, or high [18].

### Pathologic review

The resected tissues were formalin-fixed and paraffin-embedded, and stained with hematoxylin and eosin (H&E) in the Department of Pathology, Severance Hospital, Yonsei University College of Medicine. Three expert gynecologic pathologists reviewed all available H&E-stained slides obtained from IDS tissues. They independently scored each slide according to the three-tiered CRS system described by Böhm et al. [5]. Briefly, CRS is defined as follows: CRS 1: No or minimal tumor response; CRS 2: Partial tumor response: CRS 3: Complete or near-complete tumor response.

### *BRCA* testing

From the whole blood samples, genomic DNA was extracted according to the protocol provided by the manufacturer (QIAamp DNA Blood Mini Kit, QIAGEN, USA). To assess the germline mutations in *BRCA1* and *BRCA2*, the entire coding region and intron-exon boundaries of two genes were amplified using polymerase chain reaction (PCR) amplification. We identified all variants using Sanger sequencing on a 3730 DNA Analyzer with the BigDye Terminator v3.1 Cycle Sequencing Kit (Applied Biosystems, Foster City, CA, USA). Sequencing data were aligned against appropriate reference sequences (accession numbers NM_007294 and NM_000059, respectively) and analyzed using the Sequencher 5.3 software (Gene Codes Corp., Ann Arbor, MI, USA). Variations were described following the nomenclature system of the Human Genome Variation Society (http://www.hgvs.org/mutnomen) and the conventional nomenclature system from the Breast Cancer Information Core (BIC; http://research.nhgri.nih.gov/bic/). Pathogenicity interpretation of the variants were performed according to 2015 American College of Medical Genetics and Genomics guideline by professional medical geneticists, using evidences of variant type assessment, population allele frequency, prediction algorithm results, and database search such as Human Gene Mutation Database, ClinVar, and BIC.

### Statistical analysis

Descriptive data are reported as the median (range) or frequency (percentage). Categorical variables were compared with the chi-square or continuous variables with the Student’s t-test or Mann-Whitney U test for parametric/non-parametric variables, respectively. Responses were assessed according to the Response Evaluation Criteria in Solid Tumors criteria, version 1.1. We defined PFS as the time from the date of diagnosis to disease progression or death; overall survival (OS) was measured from the date of diagnosis to death or to the date of the last follow-up. Survival analysis was performed using the Kaplan-Meier method with a log-rank test. For all analyses, the significance level was set at 0.05. The statistical analyses were performed with the SPSS statistical software (version 21.0; IBM Corp., Armonk, NY).

## Results

### Patients’ characteristics

Table 1 contains a comparison of the clinical characteristics between the *BRCA* wild-type genotype group and the *BRCA* mutation group. Of the 169 patients included, 122 (71.9%) had the wild-type *BRCA* genotype, and 47 (28.1%) had the *BRCA1/2* mutations. There were no significant between-group differences in patient characteristics such as age, CA-125 level, FIGO stage, histologic type, tumor grade, tumor burden, CRS, residual disease, rate of radical surgery, surgical complexity score, chemotherapy regimen, or cycles of NAC.

**Table 1:** Patient and clinical characteristics (*N* = 169)

Characteristics	*BRCA* wild-type (*N* = 122)	*BRCA* mutation (*N* = 47)	*P*
Age, median (range), years	57 (32–77)	56 (27–76)	0.610
CA-125 level, median (range), U/mL	1958.7 (94.2.0–21,994.8)	1711.3 (75.0–23,919.0)	0.759
FIGO stage, n (%)			
III	56 (45.9%)	23 (48.9%)	0.734
IV	66 (54.4%)	24 (51.1%)	
Grading			
1	3 (2.5%)	0 (0%)	0.489
2	11 (9.0%)	2 (4.5%)	
3	102 (83.6%)	42 (89.4%)	
Not available	6 (4.9%)	3 (6.4%)	
Tumor burden assessed by diagnostic laparoscopy*			
PIV 8	27 (22.1%)	3 (6.4%)	0.097
PIV 10	25 (20.5%)	14 (29.8%)	
PIV 12	10 (8.2%)	4 (8.5%)	
PIV 14	5 (4.1%)	1 (2.1%)	
Not available	55 (45.1%)	25 (53.2%)	
CRS, n (%)			
1–2	79 (64.8%)	31 (66.0%)	0.516
3	43 (35.2%)	16 (34.0%)	
Residual disease, n (%)			
No	62 (50.8%)	18 (38.3%)	0.344
Any residual	58 (47.5%)	28 (59.6%)	
Not available	2 (1.6%)	1 (2.1%)	
Radical surgery^†^, n (%)			
None	59 (48.4%)	19 (40.4%)	0.354
Any radical surgery	63 (51.6%)	28 (59.6%)	
Surgical complexity score groups^‡^			
1–2 (Low/intermediate)	102 (83.6%)	37 (78.7%)	0.457
8 (High)	20 (16.4%)	10 (21.3%)	
Chemotherapy regimen, n (%)			
Paclitaxel + carboplatin	91 (74.6%)	34 (72.3%)	0.576
Docetaxel + carboplatin	8 (6.6%)	6 (12.8%)	
Weekly paclitaxel + carboplatin	15 (12.3%)	5 (10.6%)	
Paclitaxel + carboplatin + bevacizumab	8 (6.6%)	2 (4.3%)	

^*^ According to Fagotti et al. [15]

^†^ Radical surgery includes any of following: bowel surgery, cholecystectomy, diaphragm peritonectomy/resection, distal pancreatectomy video-assisted thoracoscopic surgery, splenectomy, liver resection, supraclavicular fossa resection, ureter resection, and others

^‡^ According to Aletti et al. [19]

CA-125, cancer antigen 125; FIGO, International Federation of Gynecology and Obstetrics; HGSC, high-grade serous carcinoma; PIV, predictive index value; CRS, chemotherapy response score

### CRS relative to *BRCA1/2* mutation status

CRS 3 patients were 43 (35.2%) and 16 (34.0%) for patients without and with *BRCA1/2* mutations, respectively (*P* = 0.516) (Table 2). Although CRS 3 rates differed between *BRCA1* (26.9%), *BRCA2* (42.9%) and the wild-type *BRCA* genotype, these difference did not achieve statistical significance.

**Table 2:** Chemotherapy response score relative to BRCA1/2 mutation status

	CRS	Wild type	Mutation	Total	*P* value
BRCA1 or 2	1–2	79 (64.8%)	31 (66.0%)	110 (65.1%)	0.516
	3	43 (35.2%)	16 (34.0%)	59 (34.9%)	
BRCA1	1–2	91 (63.6%)	19 (73.1%)	110 (65.1%)	0.243
	3	52 (36.6%)	7 (26.9%)	59 (34.9%)	
BRCA2	1–2	98 (66.2%)	12 (57.1%)	110 (65.1%)	0.280
	3	50 (33.8%)	9 (42.9%)	59 (34.9%)	

CRS, Chemotherapy response score

**Fig. 2:**
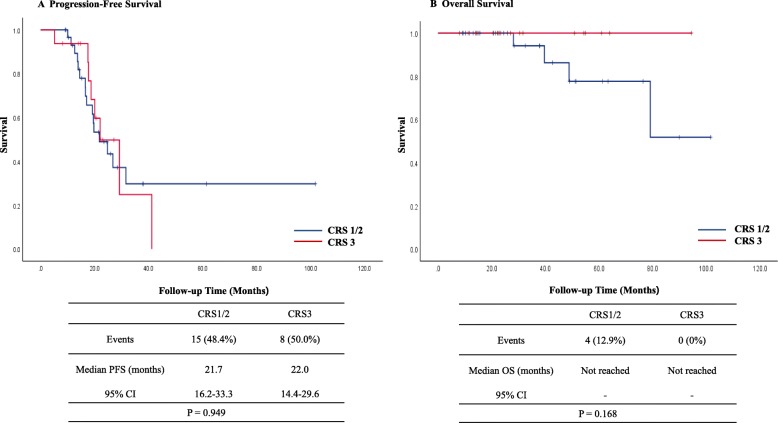
Kaplan-Meier curves of PFS (**a**) and OS (**b**) stratified by CRS in patients with *BRCA1/2* mutations. CI, confidence interval; CRS, chemotherapy response score; PFS, progression-free survival; OS, overall survival

**Fig. 3:**
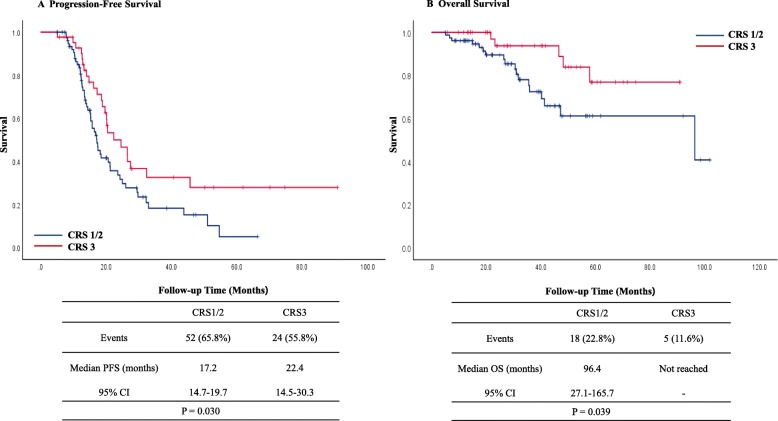
Kaplan-Meier curves of PFS (**a**) and OS (**b**) stratified by CRS in patients without *BRCA* mutations. CI, confidence interval; CRS, chemotherapy response score; PFS, progression-free survival; OS, overall survival

Kaplan–Meier curves for OS and PFS stratified by CRS in patients with the *BRCA1/2* mutations are shown in Fig. 2. Fifteen (48.4%) in the CRS 1/2 and 8 (50.0%) in the CRS 3 group had recurred by the time of the analysis. Median PFS in the CRS 1/2 group was 21.7 months (95% confidence interval [CI], 16.2–33.3) and 22.0 months (95% CI, 14.4–29.6) in the CRS 3 group. Four (12.9%) in the CRS1/2 and no patients in the CRS3 group had died by the time of the analysis. Median OS was not reached in both groups. CRS 3 in patients with the *BRCA1/2* mutations was not significantly associated with improved PFS (*P* = 0.949) and OS (*P* = 0.168). In patients without *BRCA* mutations, 52 (65.8%) in the CRS 1/2 and 24 (55.8%) in the CRS 3 group had recurred by the time of the analysis. Median PFS in the CRS 1/2 group was 17.2 months (95% CI, 14.7–19.7) and 22.4 months (95% CI, 14.5–30.3). Eighteen (22.8%) in the CRS 1/2 and 5 (11.6%) patients in the CRS 3 group had died by the time of the analysis. Median OS in the CRS 1/2 group was 96.4 months (95% C], 27.1–165.7) and not reached in the CRS 3 group. However, CRS 3 in patients without *BRCA* mutations was significantly associated with improved PFS (*P* = 0.030) and OS (*P* = 0.039) (Fig. 3). The results of the multivariate Cox regression analyses of PFS and OS in all patients are shown in Additional file 1. In terms of recurrence, multivariate analysis showed that *BRCA1/2* mutation was a marginally significant prognostic factor (HR, 0.65; 95% CI, 0.40–1.04). Multivariate analysis showed CRS 3 (HR, 0.29; 95% CI, 0.10–0.80) and *BRCA1/2* mutation (HR, 0.27; 95% CI, 0.08–0.92) were significantly associated with a longer OS.

**Fig. 4:**
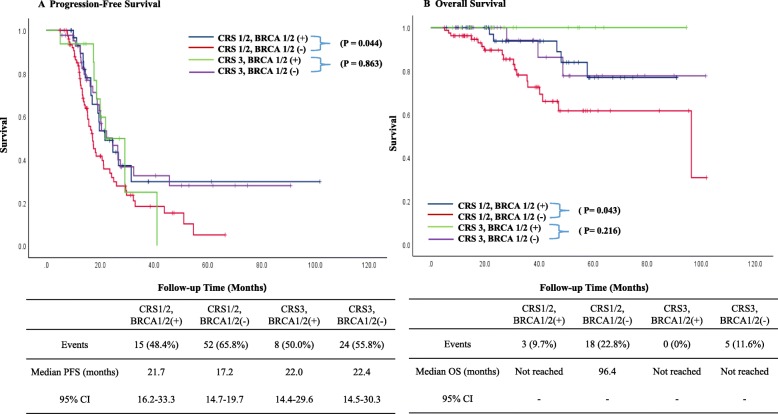
Kaplan-Meier curves of PFS (**a**) and OS (**b**) stratified by *BRCA* mutations and CRS. CI, confidence interval; CRS, chemotherapy response score; PFS, progression-free survival; OS, overall survival

We also categorized the patients based on *BRCA* mutations and CRS (*BRCA1/2* mutations with CRS 1/2; the wild-type *BRCA* genotype with CRS 1/2; *BRCA1/2* mutations with CRS 3; and the wild-type *BRCA* genotype with CRS 3) to evaluate survival according to the relationship of *BRCA1/2* mutations in the CRS. Fifteen (48.4%) in *BRCA1/2* mutations with CRS1/2, 52 (65.8%) in the wild-type *BRCA* genotype with CRS1/2, 8 (50%) in *BRCA1/2* mutations with CRS3, and 24 (55.8%) in the wild-type *BRCA* genotype with CRS3 group had recurred by the time of the analysis. Median PFS in 4 groups were 21.7 (95% CI, 16.2–33.3), 17.2 (95% CI, 14.7–19.7), 22.0 (95% CI, 14.4–29.6), and 22.4 (95% CI, 14.5–30.3), respectively. Three (9.7%) in *BRCA1/2* mutations with CRS1/2, 18 (22.8%) in the wild-type *BRCA* genotype with CRS1/2, 0 (0%) in *BRCA1/2* mutations with CRS3, and 5 (11.6%) in the wild-type *BRCA* genotype with CRS3 group had died by the time of the analysis. Median OS in the *BRCA1/2* mutations with CRS3 group was 96.4 and other 3 groups were not reached. In patients with CRS 1/2, the carriers of *BRCA1/2* mutations had better PFS (*P* = 0.044) and OS (*P* = 0.043) than the wild-type *BRCA* genotype patients. However, in patients with CRS 3, there was no significant difference in PFS (*P* = 0.863) and OS (*P* = 0.216) between *BRCA1/2* carriers and the wild-type *BRCA* genotype patients (Fig. 4). In addition, we performed a subset analysis including only in Grade 3 patients excluding the 16 patients with Grade1 or 2. Similar results were obtained for patients with Grade 3 (Additional file 2). In patients with CRS 1/2, the carriers of *BRCA1/2* mutations had better PFS (*P* = 0.015) and OS (*P* = 0.049) than the wild-type *BRCA* genotype patients. However, in patients with CRS 3, there was no significant difference in PFS (*P* = 0.917) and OS (*P* = 0.389) between *BRCA1/2* carriers and the wild-type *BRCA* genotype patients.

## Discussion

In this study, we evaluated the relationship between *BRCA1/2* mutations and CRS and survival outcomes in advanced-stage ovarian cancer patients treated with NAC followed by IDS. The patients with *BRCA1/2* mutations did not show higher CRS rates than patients without these mutations. In patients without the *BRCA1/2* mutations, the CRS 3 patients showed superior survival outcomes compared to the patients with CRS 1/2. In addition, in the CRS 1/2 patient group, the survival outcomes of the *BRCA1/2* carriers were superior to those of the wild-type *BRCA* genotype patients. In the *BRCA* wild-type group, patients who achieved CRS 1/2 after NAC had a poor prognoses compared to the patients with CRS 3. Therefore, patients with the *BRCA* wild-type should be considered for additional treatments to achieve CRS 3 after NAC, such as adding a bevacizumab or immune checkpoint inhibitors to platinum-based NAC. In addition, *BRCA* wild-type patients with CRS 1/2 after platinum-based NAC expected to have a poor prognosis due to residual tumors after NAC that may be resistant to platinum-based NAC if they receive the same NAC regimen after IDS. To improve the survival of the BRCA wild-type patients with CRS 1/2, the genomic profiles of the post-NAC tissues should be evaluated to identify biomarkers and to select targeted therapies [19].

Response rates to platinum-based NAC predict survival and may be regarded as a surrogate prognostic marker [5, 6]. A three-tiered CRS system showed a significant association with survival outcomes. The CRS system for assessing platinum-based NAC response rates can be a reproducible prognostic tool, and the incorporation of the CRS system after NAC can help determination of adjuvant treatment in advanced-stage ovarian cancer patients. Previous studies [10–12] showed that advanced-stage ovarian cancer patients with *BRCA1/2* mutations tend to have higher response rates to platinum-based chemotherapy, longer PFS, and a higher benefit from NAC. The *BRCA1/2* mutations are also being considered as specific targets. Recent randomized studies on poly (adenosine diphosphate–ribose) polymerase inhibitors have demonstrated a substantial survival benefit in *BRCA1/2* carriers [20–24], hence germline *BRCA1/2* testing is becoming a mandatory in treatment decisions in advanced-stage ovarian cancer.

In triple-negative breast cancer, several studies have shown that in the relationships between germline *BRCA1/2* mutations, response rates, and prognoses. In patients with triple-negative breast cancer, *BRCA1/2* mutation patients had higher pCR rates than those with *BRCA* wild-type patients. However, *BRCA* mutation patients do not benefit as much from a pCR compared with patients without *BRCA* mutation. In ovarian cancer patients, no such studies have yet been reported. Hahnen et al. [13] showed that triple-negative breast cancer patients without germline *BRCA1/2* mutations benefit from the addition of carboplatin and that *BRCA1/2* mutation carriers had superior response rates. Fasching et al. [14] showed the effect of *BRCA1/2* mutations in the NAC of triple-negative breast cancer. Patients with *BRCA1* or *BRCA2* mutations had higher pCR rates than patients without a mutation. However, *BRCA1/2* mutation patients who receive standard treatment do not benefit as much from a pCR as patients with the wild-type *BRCA*. These results were similar to our study.

Our study showed that patients with *BRCA1/2* mutations enjoyed a good survival outcome regardless of their CRS. In contrast, CRS 3 had a significant survival benefit on PFS and OS for wild-type *BRCA* genotype patients. In addition, the *BRCA1/2* mutations had a significant influence on survival in the CRS 1/2 patient group. One plausible explanation for the absence of the effect of CRS 3 on the prognoses for patients with the *BRCA1/2* mutations is the influence of the platinum-based adjuvant treatment after IDS. Although the CRS 1/2 group of patients was expected to have a poor prognosis, their prospects were improved if they had the *BRCA1/2* mutations because of their conferred sensitivity to platinum-based adjuvant chemotherapy after IDS. The mechanism of action by platinum drugs is mediated by the formation of the covalent binding of platinum to DNA. This binding interferes with DNA synthesis and ultimately leads to cell death [25]. It seems likely that partially formed covalent bindings cause replication of fork stalling when it is encountered by the DNA replication machinery during the S phase. These stalled replication forks may degenerate into double-stranded DNA breaks [26] in which tumor cells with defects in their DNA repair pathways achieves increased response rates against platinum drugs, as investigated in previous studies [27–30].

However, unlike in breast cancer studies, the status of *BRCA1/2* mutations in our study was unrelated to CRS. In previous studies [31, 32], patients with germline *BRCA1/2* mutations were associated with wider disease diffusion than *BRCA* wild-type patients in terms of peritoneal spread and incidence of bulky lymph nodes. Furthermore, Soslow et al. [33] showed the correlations between the genotype and characteristic morphologic appearance of ovarian cancer. The *BRCA1* mutation group had more tumor-infiltrating lymphocytes, thus suggesting that the status of *BRCA* mutations could influence the immunological microenvironment sufficiently to eventually drive the specific characteristics of disease presentation. Therefore, *BRCA1/2* mutation carriers may have a lower CRS following NAC as compared to *BRCA* wild-type patients.

The main limitation of our study was its low sample size and the small number of patients with *BRCA1/2* mutations. Although 169 patients were genotyped, there were only 47 carriers of these mutations. Second, considering the family history of patients who were diagnosed with ovarian cancer before 2017, the germline *BRCA* test was recommended for patients with a high probability of being a *BRCA1/2* mutation carrier. Olaparib treatment in the maintenance setting has been covered by Korean National Health Insurance since 2017, we have been routinely offered the germline *BRCA* test in patients diagnosed with high-grade serous ovarian cancer. In addition, because 92 people did not receive *BRCA* test during the study period, a selection bias may have influenced the outcomes. Third, NAC was only incorporated into our institution in late 2010; thus, our cohort was limited by the short follow-up period. Fourth, the type of NAC is important because CRS is affected by the response to chemotherapy. About 75% of patients received paclitaxel and carboplatin combination chemotherapy in our study, an analysis of the relationship between CRS and the type of NAC could not be conducted. \.

## Conclusion

In conclusion, in advanced-stage ovarian cancer patients treated with NAC followed by IDS, CRS 3 was not associated with increased survival for *BRCA1/2* mutation carriers than for patients without these mutations. Additional studies are needed to elucidate the effect of CRS on prognoses in advanced-stage ovarian cancer patients with and without *BRCA1/2* mutations.

## Supplementary information

**Additional file 1.** Multivariate analyses for progression-free and overall survival using a Cox proportional hazards model.

**Additional file 2.**

## Data Availability

The datasets used and analyzed during the current study are available from the corresponding author on reasonable request.

## Abbreviations

CRS: Chemotherapy response scores
IDS: Interval debulking surgery
NAC: Neoadjuvant chemotherapy
OS: Overall survival
pCR: Pathologic complete response
PCR: Polymerase chain reaction
PFS: Progression-free survival
PIV: Predictive index value
