# Supplementary material for: Germline BRCA, chemotherapy response scores, and survival in the neoadjuvant treatment of ovarian cancer
Source: BMC Cancer. 2020 Mar 4;20:185. doi: 10.1186/s12885-020-6688-8 (PMC7057666; doi:10.1186/s12885-020-6688-8)
Supplement: Supplementary file 1 — Additional file 1. Multivariate analyses for progression-free and overall survival using a Cox proportional hazards model. [file 12885_2020_6688_MOESM1_ESM.docx]

Additional file 1. Multivariate analyses for progression-free and overall survival using a Cox proportional hazards model

| Variables | PFS | | OS | |
| --- | --- | --- | --- | --- |
|  | HR (95% CI) | *P* | HR (95% CI) | *P* |
| Age, years |  |  |  |  |
| ≤57 | 1 |  | 1 |  |
| >57 | 1.01 (0.66-1.55) | 0.959 | 0.63 (0.28-1.44) | 0.276 |
| Residual disease |  |  |  |  |
| No | 1 |  | 1 |  |
| Any residual | 0.98 (0.65-1.46) | 0.904 | 1.29 (0.56-2.95) | 0.554 |
| FIGO stage |  |  |  |  |
| III | 1 |  | 1 |  |
| IV | 1.61 (1.05-2.47) | 0.031 | 0.83 (0.36-1.90) | 0.656 |
| Radical surgery^†^ |  |  |  |  |
| No | 1 |  | 1 |  |
| Any radical surgery | 1.14 (0.75-1.72) | 0.549 | 1.22 (0.54-2.76) | 0.632 |
| CRS |  |  |  |  |
| CRS 1/2 | 1 |  | 1 |  |
| CRS 3 | 0.73 (0.46-1.15) | 0.170 | 0.29 (0.10-0.80) | 0.017 |
| BRCA status |  |  |  |  |
| BRCA (-) | 1 |  | 1 |  |
| BRCA (+) | 0.65 (0.40-1.04) | 0.075 | 0.27 (0.08-0.92) | 0.036 |

CI, confidence interval; FIGO, International Federation of Gynecology and Obstetrics; HR, hazard ratio; OS, overall survival; PFS, progression-free survival;

^†^ Radical surgery includes any of following: bowel surgery, cholecystectomy, diaphragm peritonectomy/resection, distal pancreatectomy video-assisted thoracoscopic surgery, splenectomy, liver resection, supraclavicular fossa resection, ureter resection, and others.
