# Supplementary material for: Germline BRCA, chemotherapy response scores, and survival in the neoadjuvant treatment of ovarian cancer
Source: BMC Cancer. 2020 Mar 4;20:185. doi: 10.1186/s12885-020-6688-8 (PMC7057666; doi:10.1186/s12885-020-6688-8)
Supplement: Supplementary file 2 — Additional file 2. [file 12885_2020_6688_MOESM2_ESM.pdf]

A Progression-Free Survival

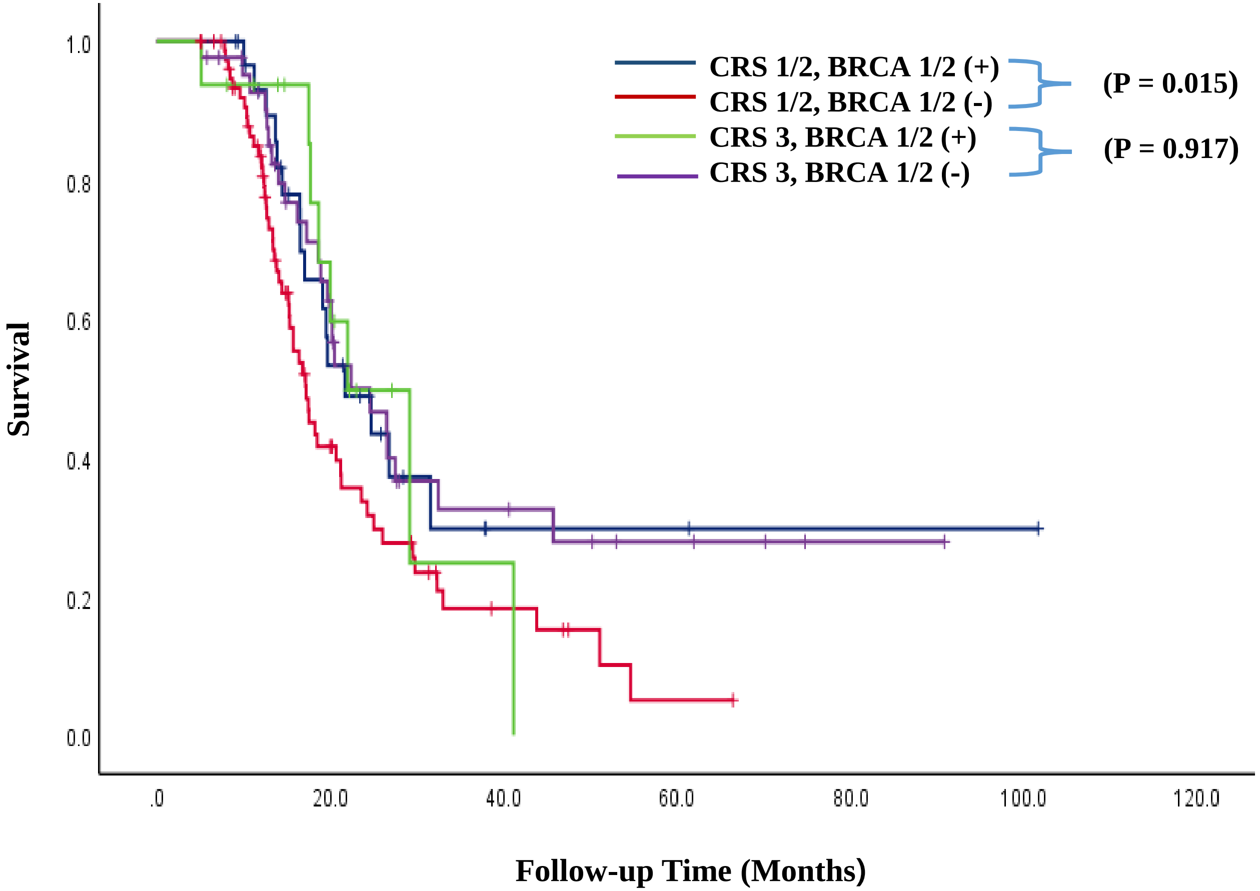

	CRS1/2, BRCA1/2(+)	CRS1/2, BRCA1/2(-)	CRS3, BRCA1/2(+)	CRS3, BRCA1/2(-)
Events	14 (46.7%)	48 (66.7%)	7 (46.7%)	19 (52.8%)
Median PFS (months)	26.8	17.5	29.2	26.5
95% CI	21.1-32.5	14.6-20.3	19.4-38.9	17.1-3

B Overall Survival

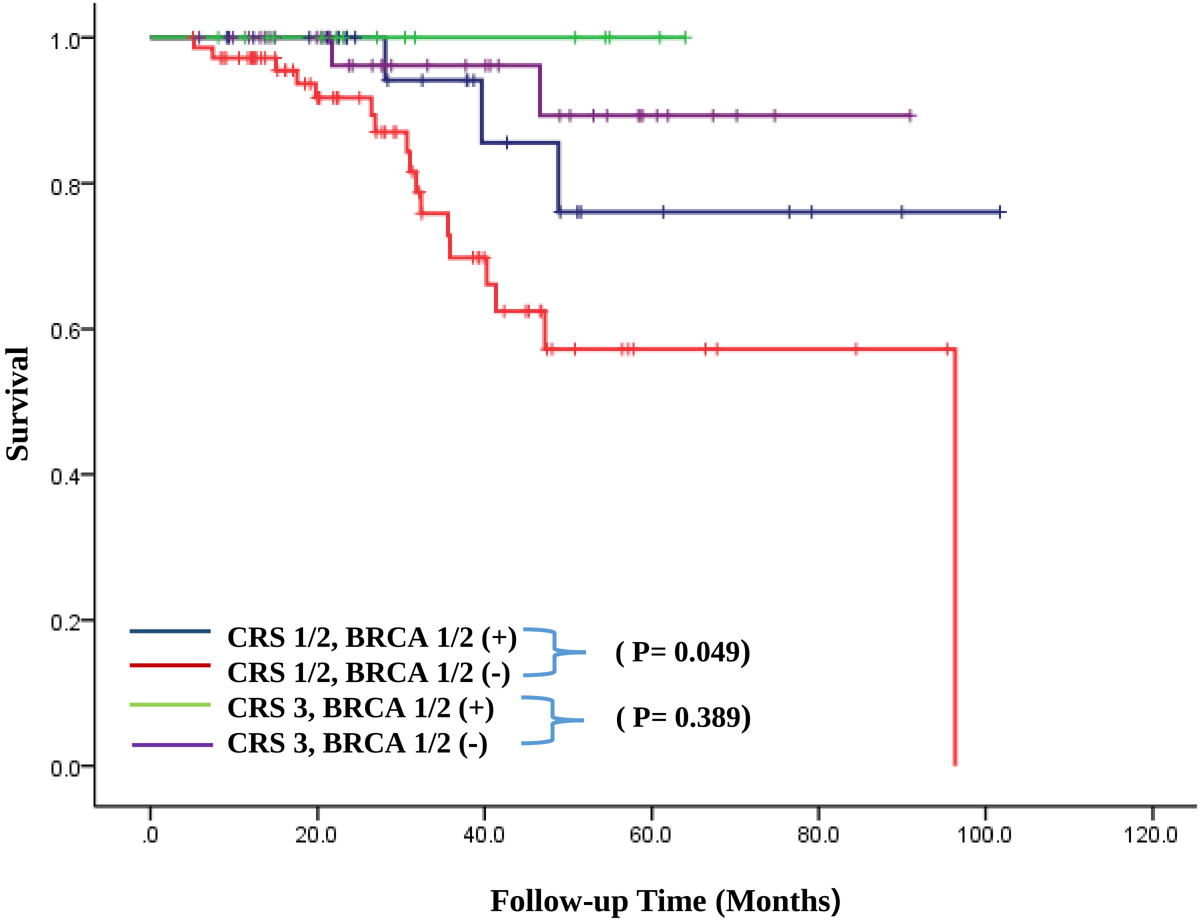

	CRS1/2, BRCA1/2(+)	CRS1/2, BRCA1/2(-)	CRS3, BRCA1/2(+)	CRS3, BRCA1/2(-)
Events	3 (10.0%)	17 (23.6%)	0 (0%)	2 (5.6%)
Median OS (months)	Not reached	96.4	Not reached	Not reached
95% CI	-	-	-	-
